# Update on the First Finding of the Rat Lungworm, *Angiostrongylus cantonensis,* in *Rattus* spp. in Continental Europe, Valencia, Spain, 2022

**DOI:** 10.3390/pathogens12040567

**Published:** 2023-04-06

**Authors:** María Teresa Galán-Puchades, Mercedes Gómez-Samblás, Antonio Osuna, Sandra Sáez-Durán, Rubén Bueno-Marí, Màrius V. Fuentes

**Affiliations:** 1 Parasites & Health Research Group, Department of Pharmacy, Pharmaceutical Technology and Parasitology, Faculty of Pharmacy, University of Valencia, 46100 Valencia, Spain; sandra.saez@uv.es (S.S.-D.); ruben.bueno@uv.es (R.B.-M.); mario.v.fuentes@uv.es (M.V.F.); 2 Laboratory of Biochemistry and Molecular Parasitology, Institute of Biotechnology, University of Granada, 18071 Granada, Spain; msambla@gmail.com (M.G.-S.); aosuna@ugr.es (A.O.); 3 Laboratorios Lokímica, Departamento de Investigación y Desarrollo (I+D), Ronda Auguste y Louis Lumière 23, Nave 10, Parque Tecnológico, Paterna, 46980 Valencia, Spain

**Keywords:** *Angiostrongylus cantonensis*, *Rattus norvegicus*, *Rattus rattus*, Valencia, orchards, Spain

## Abstract

The rat lungworm, *Angiostrongylus cantonensis*, is an emerging parasite that can cause eosinophilic meningitis in humans. Over the past 60 years, the worm has greatly expanded its original Asian distribution to most tropical and subtropical areas of the world, mostly by traveling on ships with its definitive hosts, rats. *Angiostrongylus cantonensis* was recently found for the first time in Continental Europe, specifically in 3 (2 *Rattus norvegicus* and 1 *Rattus rattus*) out of 27 rats trapped in the sewer system of the city of Valencia, Spain. Updating the investigation, the parasite has subsequently been found in 8 (5 *R. norvegicus* and 3 *R. rattus*) out of 94 rats analyzed. The highest prevalence of infection (20%) was obtained in rats trapped in the orchards that surround the city, where both snails and slugs (intermediate hosts) abound, and where vegetables consumed in Valencia, other parts of Spain, and abroad, are produced. The presence of the parasite in rats does not necessarily mean that the disease it causes becomes a relevant public health concern since it strongly depends on the food habits of the population at risk. If proper precautions are taken, the risk of acquiring neuroangiostrongylosis should be minimal.

## 1. Introduction

*Angiostrongylus cantonensis* is a zoonotic pathogen parasite of the pulmonary arteries of rats, mainly of the sewer rat, *Rattus norvegicus,* and the black rat, *Rattus rattus.* The rat lungworm was first described in Chinese rats in 1935 [1]. Its life cycle was elucidated in Brisbane (Australia) almost 70 years ago [2,3]. Briefly, adult nematodes live in the pulmonary arteries of rats in which first-stage larvae, hatched from the eggs laid by females, migrate up the bronchial tree and are then swallowed and later excreted in rat feces. Snails and slugs act as intermediate hosts, and a variety of paratenic hosts (freshwater shrimp, crabs, and mollusks) can also harbor the third-stage infective larvae [4]. Rats become infected after the ingestion of these L3 larvae, which migrate to the central nervous system (CNS) and turn into young adults, which, in turn, reach maturity in the pulmonary arteries.

Humans can become infected by *A. cantonensis* when—as in the case of rats—they accidentally ingest the L3 larvae. Consequently, angiostrongylosis is a food-borne disease related to the ingestion of raw or undercooked intermediate or paratenic hosts. Likewise, infective larvae can contaminate fresh vegetables, which, at some point, were in contact with infected intermediate hosts, as the L3 larvae are excreted in snail/slug slime [5]. Therefore, humans could also become infected by eating, for example, salads containing fresh vegetables, such as lettuce, lamb’s lettuce, arugula, etc., with traces of infective slime [6].

Humans are not suitable definitive hosts for the parasite, so the ingested migrating larvae usually end up dying in the CNS. The presence of these nematodes in the brain can cause eosinophilic meningitis. In fact, *A. cantonensis* is one of the most common pathogens causing this disease (neuroangiostrongylosis). In addition to eosinophilic meningitis, other human conditions, such as abdominal pain of parasitic origin [7], ascending paralysis [8], and small fiber neuropathy [9], have also (sporadically) been attributed to *A. cantonensis*. Since the first case of human eosinophilic meningitis, reported in Taiwan in 1945 [10], around 3000 cases (suggested to be around 7000) have been documented in the literature [11,12].

The parasite, generally accepted as native to Southeast Asia, has spread to more than 30 countries during the last 60 years. Globalization has favored its appearance in other parts of the world, such as Japan, the Pacific Islands, Australia, the Caribbean, the USA, South America, and Africa [12]. The 22 cases of eosinophilic meningitis reported in Europe by 2020 were of imported origin, mainly from French Polynesia, Southeast Asia, and the Caribbean Islands [13]. Only one immunologically diagnosed case in France had no travel history [14].

In Europe, *A. cantonensis* has been found exclusively at insular levels, specifically in rats in Tenerife (Canary Islands) [15] and in hedgehogs in Mallorca (Balearic Islands) [16]. We recently reported the first autochthonous cases of *A. cantonensis* in *R. norvegicus* and *R. rattus* in Continental Europe, particularly in the city of Valencia, Spain, in 2021 [17]. We found the parasite in two *R. norvegicus* and one *R. rattus* among the first batch of 27 rats trapped in the sewer system of Valencia. Continuing with these studies on parasitic zoonoses in rats, in this update, we report, show in images, and describe the presence of *A. cantonensis,* not only in rats from the sewer system but also from city parks and orchards located in the peri-urban area of Valencia.

## 2. Materials and Methods

### 2.1. Study Area and Animals

**Figure 1:**
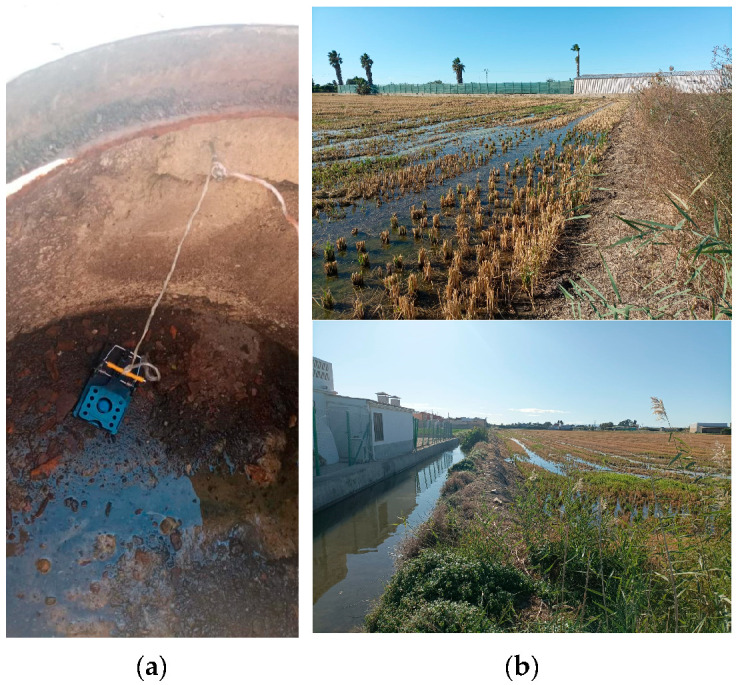
Study areas: (**a**) Detail of a trap located in the sewer system; (**b**) two views of the orchards where infected rats were trapped.

In total, 94 rats were studied, specifically, 73 *R. norvegicus* (29 males, 39 females, and 5 indeterminate) and 21 *R. rattus* (8 males and 13 females). The identification of the rat species was made based on external morphometry according to J. Gosálbez [18]. The study area comprised the sewer system of Valencia (33 *R. norvegicus* and 3 *R. rattus*), parks located in residential neighborhoods of the city (25 *R. norvegicus* and 9 *R. rattus*), and an orchard located in the peri-urban area of the city (15 *R. norvegicus* and 9 *R. rattus*) (Figure 1). Snap traps were located from April 2021 to June 2022 by the pest control company Laboratorios Lokímica, which had been commissioned with the task by the Pest Control Section of the Health Service of Valencia City Council. The main criterion for selecting the sampling points was related to previous evidence of rat activity in the area since the rat surveillance and control program is implemented by the municipality throughout the year.

The trapped rats were preserved at −20 °C until their parasitological study by the Parasite & Health Research Group of the University of Valencia. The data from the individuals (species identification, weight, and morphometry) were collected before dissection. According to their body weight and external morphometry, 47 sewer rats were considered adults (≥150 g) and 26 juveniles (<150 g). In the case of the black rats, 16 were considered adults (≥100 g) and 5 juveniles (<100 g) [18].

### 2.2. Parasitological and Molecular Techniques

The rat organs were placed in Petri dishes and examined under a dissecting microscope (Meiji Techno EMT Stereo Microscope) searching for helminth parasites. Several nematodes extracted from the heart and lung arteries were frozen at −80 °C, while other specimens were preserved in 70% ethanol until their final study. For their morphological identification, the nematodes were studied by direct examination between the slide and cover slip with lactophenol as the clearing fluid.

To confirm the species identification, total genomic DNA was isolated by using the DNeasy Blood and Tissue Kit (QIAGEN, https://www.qiagen.comExternal Link, accessed on 12 January 2023) according to the manufacturer’s instructions. The nematode species identity was confirmed by PCR and sequencing of the cytochrome c oxidase subunit 1, as well as the second internal transcribed spacer region [17]. The sequences were quality trimmed, aligned, and analyzed by Geneious software [19] for the phylogenetic tree preparation. The same software was used to compute the corrected (HKY) pairwise genetic distances among the haplotypes, and the neighbor-joining method was used for building the phylogenetic tree.

### 2.3. Statistical Analysis

For the *A. cantonensis* adults, the number of parasitized hosts, prevalence, mean intensity, mean abundance, and range of parasitization were analyzed according to Bush et al. [20]

The influence of the intrinsic (age and sex) and extrinsic (site of capture) factors related to the rats on the prevalence, mean intensity, and mean abundance of *A. cantonensis* in both rat species was assessed by the analysis of these parasitological parameters through the χ^2^ test to compare the prevalence, and the non-parametric Mann–Whitney test and the Kruskal–Wallis test to compare the mean intensity and mean abundance, respectively.

Statistical significance was established at *p* < 0.05. Statistical analyses were carried out using the IBM SPSS 26.0 for Windows (International Business Machines Corporation (IBM), Armonk, NY, USA) and StatView 5.0 (Statistical Analysis System (SAS) Institute Inc., Cary, NC, USA) software packages.

### 2.4. Scanning Electron Microscopy

Male and female specimens of the isolated nematodes from the pulmonary arteries were fixed for 12 h in a 2.5% glutaraldehyde solution in 0.05 M of cacodylate buffer (CB), pH = 7.4, plus 0.1 M of sucrose in order to obtain an isotonic fixative solution at 4 °C, washed three times in 0.1 M of CB at 4 °C and post-fixed for 2 h in 2% osmium tetroxide in 0.1 M of CB at room temperature. After the worms were washed in the same buffer, they were dehydrated for 45 min in a graded series of ethanol at room temperature (15 min changes each time). Subsequently, the nematodes were desiccated using a critical point dryer (LEICA EM CPD 300), then evaporated with carbon using an EMITECH K975X, and examined under a ZEISS Supra 40VP high-resolution variable-pressure SEM.

## 3. Results

**Figure 2:**
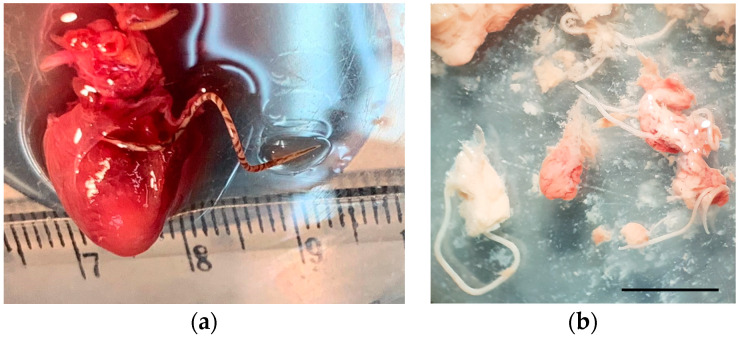
*Angiostrongylus cantonensis*: (**a**) Adult nematode emerging from inside the pulmonary artery of an infected *Rattus norvegicus*; (**b**) subadults in the brain of an individual of *Rattus rattus* (scale bar: 500 μm).

Five *R. norvegicus* as well as three *R. rattus* were infected with adult nematodes located in the heart and lung arteries, which, after their morphological and molecular study, were identified as *A. cantonensis* (Figure 2a). Likewise, one of the infected *R. rattus* also presented subadult stages in the brain (Figure 2b).

**Figure 3:**
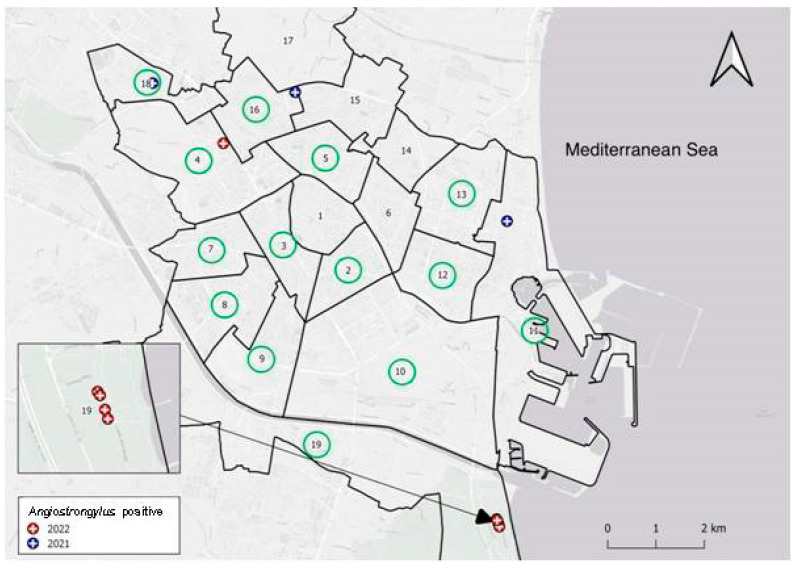
Map of the districts of Valencia; green circles showing the studied ones. *Angiostrongylus cantonensis*-positive rats were captured in the sewer system of districts with blue circles (captures in 2021). Red circles indicate captures in 2022, in a park (District No. 4) and in the orchards (No. 19).

Valencia is administratively divided into 19 districts. Figure 3 shows the location of the 14 districts where the 94 rats were trapped and the 5 in which the infected rats were found.

### 3.1. Helminthological Analysis

The overall prevalence in the 94 rats studied was 8.51% (8 infected rats out of 94 individuals), 6.85% in *R. norvegicus* (5/73), and 14.29% (3/21) in *R. rattus*. Table 1 shows the occurrence of the rat lungworm according to the data (species, sex, and age) of the infected rats trapped at the different sites (sewer system, parks, and orchards) of Valencia. The only statistically significant differences found were those concerning the sex of the rat, with the *A. cantonensis* prevalence being higher in males than in females (χ^2^ = 7.633; *p* = 0.0057).

Considering both rat species, the highest *A. cantonensis* prevalence (20%) was found in adult male rats in the orchards (30% in the *R. norvegicus* and 20% in the *R. rattus*) (Table 1). In the sewer system, the prevalence was 8.3%, and the lowest one was obtained in the parks of the city (2.94%).

Specifically, 74 *A. cantonensis* individuals were found in the eight infected rats, which represents an overall mean intensity of 9.25 (SE = 3.23; range = 2–30) and an overall mean abundance of 0.79 (SE = 0.37). In total, 67 adults (27 males and 40 females) were found in the rats, 32 (16 males and 16 females) in the five infected sewer rats, and 35 adults (11 males and 24 females) and seven subadults (1 male and 6 females) in the parasitized black rats. No statistically significant differences were found concerning any of the analyzed variables.

### 3.2. Morphology and Morphometry

**Figure 4:**
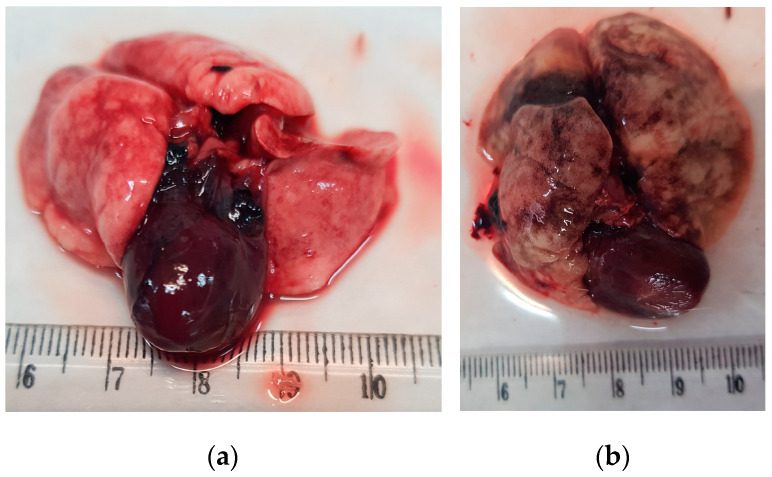
Macroscopic lesions of infected rat lungs: (**a**) White areas in the lung lobes as well as cardiomegaly in an individual of *Rattus rattus*; (**b**) white to grey areas in the lung lobes of a *Rattus norvegicus* specimen.

Macroscopically, the lungs of the infected rats showed white areas with atelectasis or emphysema (Figure 4). Patent cardiomegaly was also observed in some heavily infected pulmonary arteries or/and the heart of specific individuals (Figure 4a).

**Figure 5:**
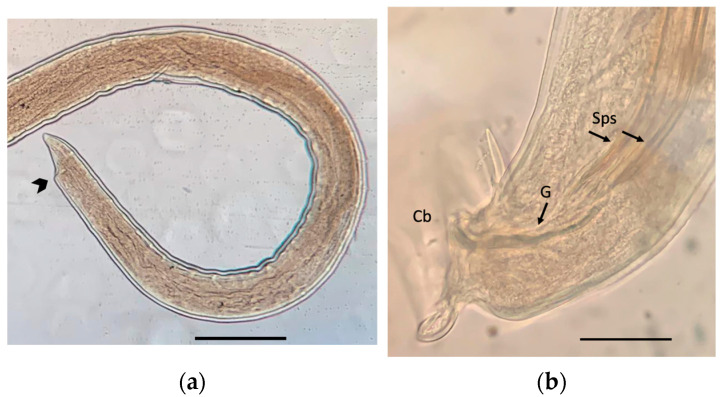
*Angiostrongylus cantonensis* subadults: (**a**) Posterior end of a female, arrow points at the vulva (scale bar 500 μm); (**b**) posterior end of a male showing the two spicules (Sps), the gubernaculum (G), and the copulatory bursa (Cb) (scale bar: 100 μm).

Figure 5 shows views of two subadults, male and female, from the brain of an infected *R. rattus.* The total length of the subadults varied between 6.60 and 13.30 mm (mean 10.47 mm) with a maximum width of 0.22 mm (0.18–0.25 mm). The spicula of the male subadult and the gubernaculum measured 1.76 mm and 0.10 mm in length, respectively.

**Figure 6:**
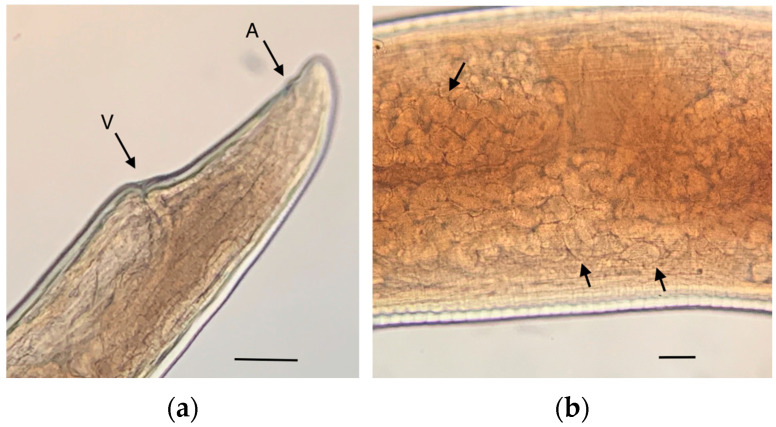
*Angiostrongylus cantonensis* adult female: (**a**) Posterior end showing the vulva (V) and the anus (A); (**b**) portion of the uterus containing the eggs (arrows). Scale bars: 50 μm.

Figure 6 shows the details of the adult female morphology. Figure 6b shows a portion of the uterus of a female containing eggs, which were measured inside the uterus of several females and were 66.46 (60.72–78.40) × 35.82 (29.40–40) μm in size (n = 50).

**Figure 7:**
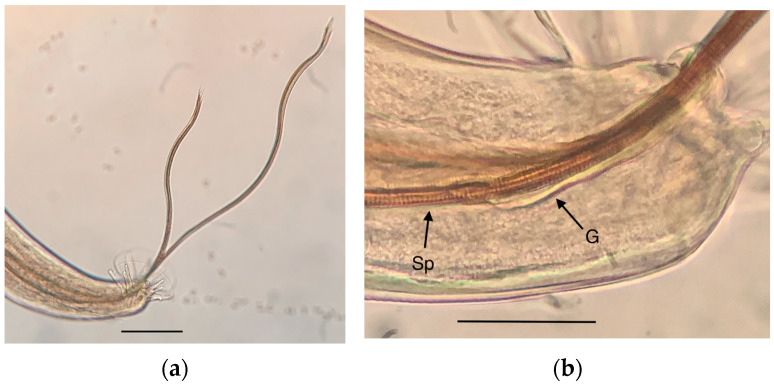
*Angiostrongylus cantonensis* adult male: (**a**) Posterior end showing the copulatory bursa and the spicules; (**b**) detail of the striated spicula (Sp) and the gubernaculum (G). Scale bars: 50 μm.

Figure 7 shows the copulatory bursa, the rays, and the two subequal and flexible spicules (Figure 7a), and a detail of the striated morphology of the approx. 10 μm wide spicules (Figure 7b).

Table 2 lists the measurements of both the male and female individuals of the rat lungworms.

**Figure 8:**
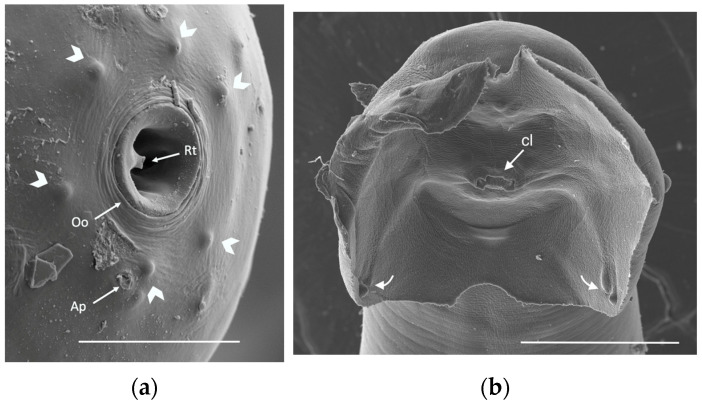
SEM images of *Angiostrongylus cantonensis* adult stages: (**a**) Anterior end of a female showing six cephalic papillae (short arrows) and an amphidial pore (ap) surrounding the oral opening (Oo) that shows a single rudimentary rectangular tooth (Rt) (scale bar 10 μm); (**b**) posterior end of a male showing a ventral view of the copulatory bursa. Arrows pointed at the cloaca (cl) and papilliform structures in terminal sections of the rays (scale bar: 50 μm).

Figure 8 shows two images by scanning electron microscopy (SEM) of the anterior end of an adult female and the posterior end of an adult male of *A. cantonensis.*

### 3.3. Molecular Analysis

Eight nematode individuals (from both *R. norvegicus* and *R. rattus* trapped in the orchards) were identified by PCR (Figure S1) and sequencing of the second partial region of the internal transcribed spacer (ITS-2) and partial cytochrome c oxidase subunit 1 gene (COI). These sequences were clustered and used for phylogenetic analyses (GenBank accession numbers OQ371987-OQ371994, OQ359781-OQ359774).

The phylogenetic tree based on the ITS2 sequences of Angiostrongylus is shown in Figure S2. All *A. cantonensis* sequences, including ours, fell into a well-supported clade separate from *A. malaysiensis*, *A. costaricensis*, *A. vasorum*, *A. chabaudi*, and *A. daskalovi*.

A phylogenetic analysis, based on the COI sequences (Figure S3), revealed clear differences among the *A. cantonensis* isolates, forming the sequences of the present study into two groups. Two male individuals were close to the *A. cantonensis* isolated from the USA, Tenerife, Mallorca, or Valencia (our previous study [17]), and six samples, four females and two males, were clustered close to the *A. cantonensis* isolated from Brazil (pairwise p-distances among the COI sequences from the *A. cantonensis* isolated individuals are shown in Table S1). Figure S4 shows the alignment with a nucleotide variation for the RV85_male and RV85_male sequences with respect to the RV85_female, RV78_female, RV78_male, RV76_male, and RV76_female sequences.

## 4. Discussion

The first finding of *A. cantonensis* in rats, both in *R. norvegicus* and *R. rattus* in Continental Europe, supports the emerging character of the parasite, which has frequently been pointed out in the literature over recent years [12]. In addition, neuroangiostrongylosis is also considered a neglected disease [12,21,22].

As mentioned before, the rat lungworm is considered an endemic parasite of tropical and subtropical parts of the world. The global model for the present distribution of the parasite predicts precisely that the most suitable habitat for the parasite is located near the equator in tropical to subtropical regions [23]. However, the potential future distribution of *A. cantonensis,* based on climate change RCP (Representative Concentration Pathways) scenarios, predict a shift away from the equator to the north and east of the Northern Hemisphere by the 2050s [23]. All the models analyzed suggested an increase in suitable habitats for *A. cantonensis* in Europe. The findings of the rat lungworms in the Balearic Island of Mallorca and in Valencia are consistent with this prediction of the future distribution of the parasite.

Nevertheless, those areas currently considered non-endemic (as European countries seem to be) may well include countries where no investigation has taken place, and, therefore, cannot be considered free from *A. cantonensis* infection [24]. In this sense, the scarcity of surveys investigating the helminth parasites of rats in cities in developed countries is rather surprising [25].

The success of the spread of *A. cantonensis* resides in the sheer diversity of its intermediate hosts and the efficient dispersion of ship-borne rats [26]. The current distribution of the parasite, based on data extracted from the literature [12], shows that most of the affected localities are situated on islands or on the coasts of the affected countries, consistent with the point of entry of the parasite.

**Figure 9:**
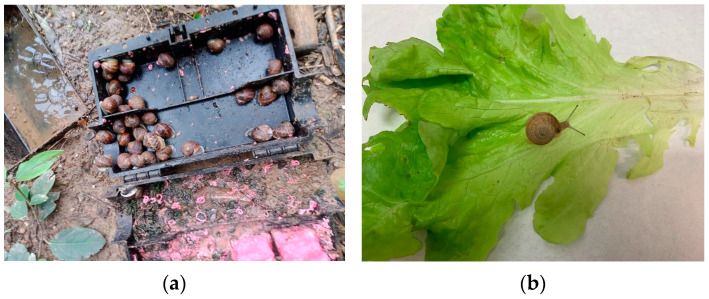
(**a**) Snails found in a box containing rat baits after 24 h placed in the orchards investigated. (**b**) Snail found on lettuce purchased in a market in Valencia, coming from an organic orchard.

In this context, Valencia is the second Spanish maritime port in traffic, with direct connections to endemic countries [27]. However, once on land, the parasite must find a suitable malacological fauna to serve as an intermediate host. At this point, it is relevant to highlight the fact that the city of Valencia is surrounded by orchards. The 1200-year-old “Horta of Valencia“ was recognized on the register of Globally Important Agriculture Heritage Systems (GIAHS) by the Food and Agricultural Organization (FAO) of the United Nations (https://www.fao.org/newsroom/detail/Spain-s-Horta-of-Valencia-wins-recognition-on-FAO-s-global-agricultural-heritage-list/en, accessed on 19 December 2022). In the Valencian orchards, where a variety of vegetables is produced, snails and slugs abound (Figure 9a), especially in ecological (organic) orchards in which pesticides (including molluscicides) are not used (Figure 9b). As a result, the prevalence of *A. cantonensis* was the highest in the orchards (Table 1), located near the port of Valencia (District No. 19) (Figure 3), the likely point of entry of the parasite.

It is worth mentioning that in two rats trapped in the orchards (one *R. norvegicus* and the other *R. rattus*), two different *A. cantonensis* sequences were found in the same individual (Figure S3), one close to those from the USA and the other closer to isolates from Brazil, indicating different routes of parasite colonization from different endemic localities.

The fact that several rats studied in the orchards (including one of the individuals infected by *A. cantonensis*) also harbored intestinal trematodes (unpublished data), whose intermediate hosts are also snails, indicates the presence of a suitable malacofauna for both nematodes and trematodes. The presence of *A. cantonensis* is currently being investigated in snail/slug individuals coming from the districts of Valencia in which the parasite was found in order to determine the nature of its intermediate hosts.

When the parasite is found in rats, the parasite is deemed to be endemic in that region [24]. However, it does not mean that the disease it causes, eosinophilic meningitis, becomes a relevant public health concern since it strongly depends on the food habits of the population at risk. Although a sporadic case of autochthonous eosinophilic meningitis was reported in Spain that could not be confirmed but was suggested to be caused by *A. cantonensis* [28], there have been no confirmed cases of neuroangiostrongylosis in Spain.

Generally speaking, and excluding any exceptions that may occur, it is not customary in Spain to eat either the intermediate hosts or paratenic ones raw or undercooked. Snails are commonly consumed in Valencia (and other parts of Spain), but always well cooked in dishes such as “Caragolà” (snail stew), or as an ingredient in the well-known “Valencian paella”, rendering these boiled snails non-infectious. However, people who gather the mollusks in the wild or workers who handle them in snail farms could be at risk of contracting the disease from dirty hands that, at some point, have come into contact with infective snail slime.

The Mediterranean diet is characterized by the regular consumption of fruits and vegetables. Considering that the infective L3 larva can survive up to 72 h in slime [29,30], ingesting poorly washed vegetables could be a feasible route of human infection, as well as the accidental ingestion of the remains of infected snails/slugs in vegetable juices [31]. In fact, samples of lettuces purchased from Kuala Lumpur (Malaysia) public markets showed small numbers of living, infective L3 larvae of *A. cantonensis* (two to three larvae per 50 g of lettuce leaf) [29]. Therefore, considering that the vegetables produced in the “Horta of Valencia” are not only consumed in Valencia but also in other parts of Spain, and even exported to other countries, although minimal, there is the likelihood that consumers could become infected outside of Valencia.

The role of these L3 larvae contaminating gastropod slime as an effective mode of transmission to humans is controversial, as the different results obtained on the number of larvae that can be released into intermediate host slime appear not to be sufficiently consistent [32]. Apparently, the phenomenon of spontaneous shedding of larvae may be restricted to heavily infected gastropods [29]. Notwithstanding, large numbers of L3 have been observed emerging from dead or dying snails [12,29]. Consequently, it should be considered, first, that handling (recently) deceased snails could pose a higher risk than manipulating live ones. Secondly, the use of molluscicides does not ensure the absence of infective larvae in vegetables, since, by escaping from the dying snails, they could contaminate the vegetables for some time. Therefore, this route of transmission by released larvae present in vegetables or dirty hands should not be ruled out.

## 5. Conclusions

The finding of *A. cantonensis* in rats in Valencia is of public health concern. However, if proper precautions are taken, the risk of acquiring neuroangiostrongylosis should be minimal. The Health Department of the Valencian regional government was duly informed of the finding and, consequently, more exhaustive surveillance of rats is to take place, not only in the city but also in the orchards, while the budget allocated to the control measures has been increased.

Likewise, the Spanish medical community (particularly that of Valencia) should be aware of the existence of neuroangiostrongylosis and, from now on, *A. cantonensis* should be included, like other bacterial or viral pathogens, in the differential diagnosis of patients with symptoms compatible with meningitis, not only in those with a travel history to endemic countries but also in those who have not left Spain.

Considering the aforementioned importance of the activity of the port of Valencia, with direct connections to endemic countries, and also taking into account its continuous connections with the port of Mallorca, it is likely that some infected rats arrived at some point on this island, where the parasite was found in 2019 [16] and from which Valencia is only 260 km away.

Further studies are scheduled to, in addition to determining the intermediate hosts of the parasite, find out the true extent of rat lungworm in Valencia and its surroundings. The fact that the parasite was found not only near the port of Valencia but also in rats trapped about 10 km from the port (Figure 3, District No. 18) seems to indicate that *A. cantonensis*, which most likely entered through the port from different endemic countries —as suggested by the different sequences found in the parasites—has successfully spread over the years.

## Figures and Tables

**Table 1:** Occurrence of *Angiostrongylus cantonensis* in *Rattus* spp. trapped in Valencia, Spain, in 2021–2022.

Trapping Sites	*Rattus* *norvegicus*	*Rattus rattus*
	*p* (%) (+/n)	*p* (%) (+/n)
Sewer system	6.90 (2/33)	33.33 (1/3)
Males	16.66 (2/12)	0 (0/0)
Females	0 (0/18)	33.33 (1/3)
Indet. * sex	0 (0/3)	---
Adults	9.52 (2/21)	0 (0/1)
Juveniles	0 (0/12)	50 (1/2)
Parks	0 (0/25)	11.11 (1/9)
Males	0 (0/7)	33.33 (1/3)
Females	0 (0/17)	0 (0/6)
Indet. * sex	0 (0/1)	---
Adults	0 (0/14)	14.28 (1/7)
Juveniles	0 (0/11)	0 (0/2)
Orchards	20 (3/15)	11.11 (1/9)
Males	30 (3/10)	20 (1/5)
Females	0 (0/4)	0 (0/4)
Indet. * sex	0 (0/1)	---
Adults	25 (3/12)	12.50 (1/8)
Juveniles	0 (0/3)	0 (0/1)
TOTAL	6.85 (5/73)	14.29 (3/21)

* Indeterminate.

**Table 2:** Measurements of 12 males and 10 females of *Angiostrongylus cantonensis* collected from rats trapped in the orchards of Valencia, Spain, 2022.

Morphological Character (in mm)	Males (n = 12) Mean (Range)	Females (n = 10) Mean (Range)
Total length	19.29 (14.08–23.63)	25.98 (14.83–32.68)
Maximum width	0.34 (0.25–0.45)	0.50 (0.25–0.63)
Esophagus length	0.31 (0.26–0.36)	0.35 (0.29–0.39)
Esophagus maximum width	0.09 (0.07–0.11)	0.10 (0.07–0.15)
Distance from excretory pore to cephalic extremity	0.39 (0.29–0.47)	0.36 (0.19–0.43)
Spicule length	1.24 (1.03–1.47)	-
Gubernaculum length	0.11 (0.09–0.15)	-
Distance from vulva to posterior end of the body	-	0.21 (0.18–0.25)
Distance from anus to posterior end of body	-	0.07 (0.05–0.10)
Distance from vulva to anus	-	0.14 (0.11–0.18)
Vulva region width	-	0.17 (0.14–0.23)
Anus region width	-	0.09 (0.06–0.12)

## Data Availability

Not applicable.

## Supplementary Materials

The following supporting information can be downloaded at: https://www.mdpi.com/article/10.3390/pathogens12040567/s1, Figure S1: 1% agarose gel. M; hyperladder I; Figure S2: Neighbor-Joining phylogenetic tree of *Angiostrongylus cantonensis*, *A. malaysiensis*, *A. costaricensis*, *A. vasorum*, *A. chabaudi*, and *A. daskalovi* based on the partial ITS2 sequences; Figure S3: Neighbor-Joining phylogenetic tree of *Angiostrongylus cantonensis* geographical isolated individuals based on the partial COI sequences; Figure S4: ClustalW alignment performed with partial COI sequences; Table S1: Pairwise p-distance between the COI sequences from *Angiostrongylus cantonensis* isolated individuals.
