# Supplementary material for: Update on the First Finding of the Rat Lungworm, Angiostrongylus cantonensis, in Rattus spp. in Continental Europe, Valencia, Spain, 2022
Source: Pathogens. 2023 Apr 6;12(4):567. doi: 10.3390/pathogens12040567 (PMC10143490; doi:10.3390/pathogens12040567)
Supplement: Supplementary file 1 [file pathogens-12-00567-s001.zip › Figures S1–S4.pdf]

Figures S1–S4

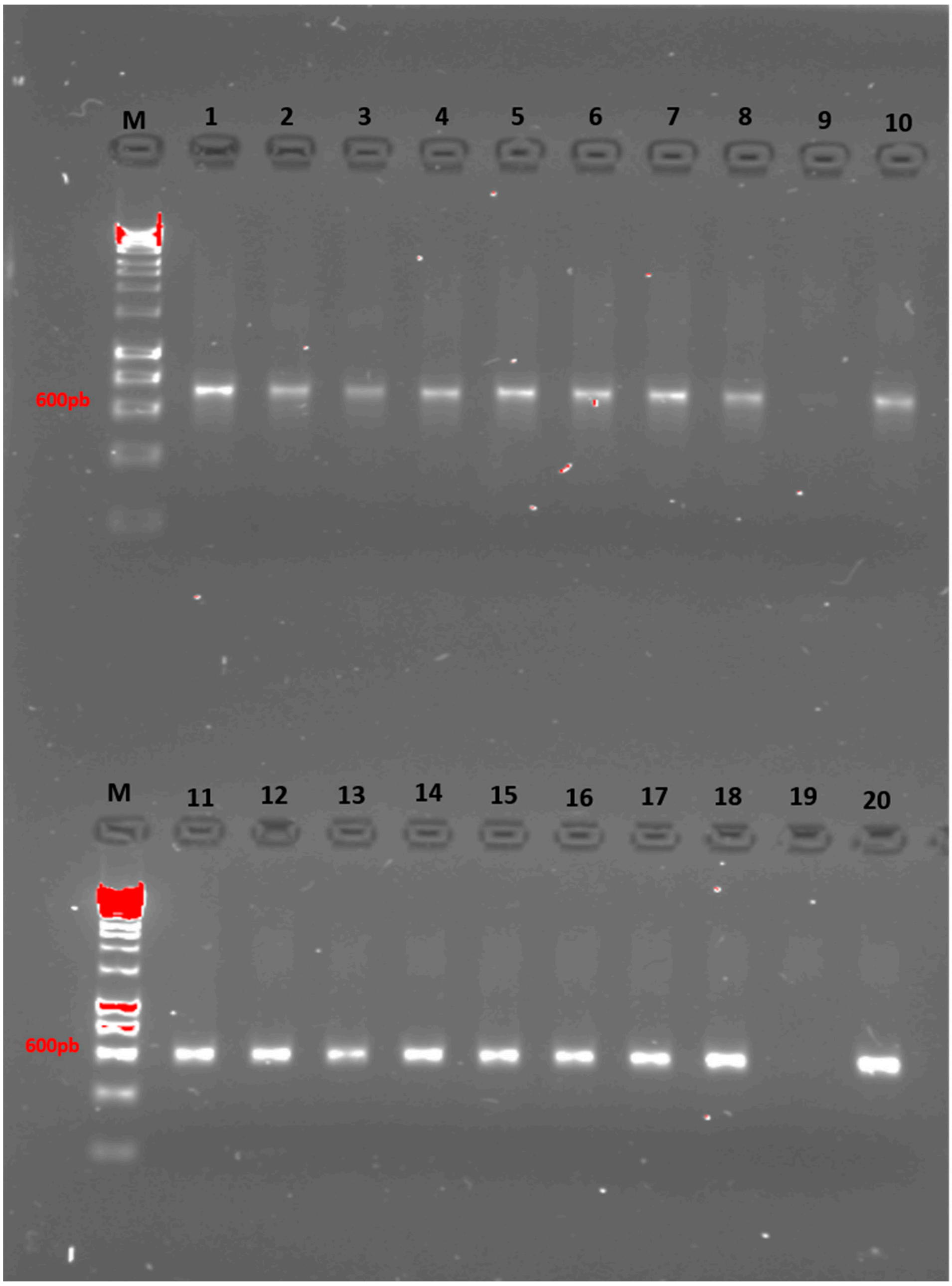

Figure S1. 1% agarose gel. M; hyperladder I. From line 1 to line 10 the amplicon corresponds to the COI gene and the follow samples respectively: RV76 female, RV76 male, RV83 female, RV78 female, RV78 male, RV85 male, RV83 male, RV85 female, negative control, positive control. From line 11 to 20 the amplicon corresponds to the ITS-2 gene and the follow samples respectively: RV76 female, RV76 male, RV83 female, RV78 female, RV78 male, RV85 male, RV83 male, RV85 female, negative control, positive control.

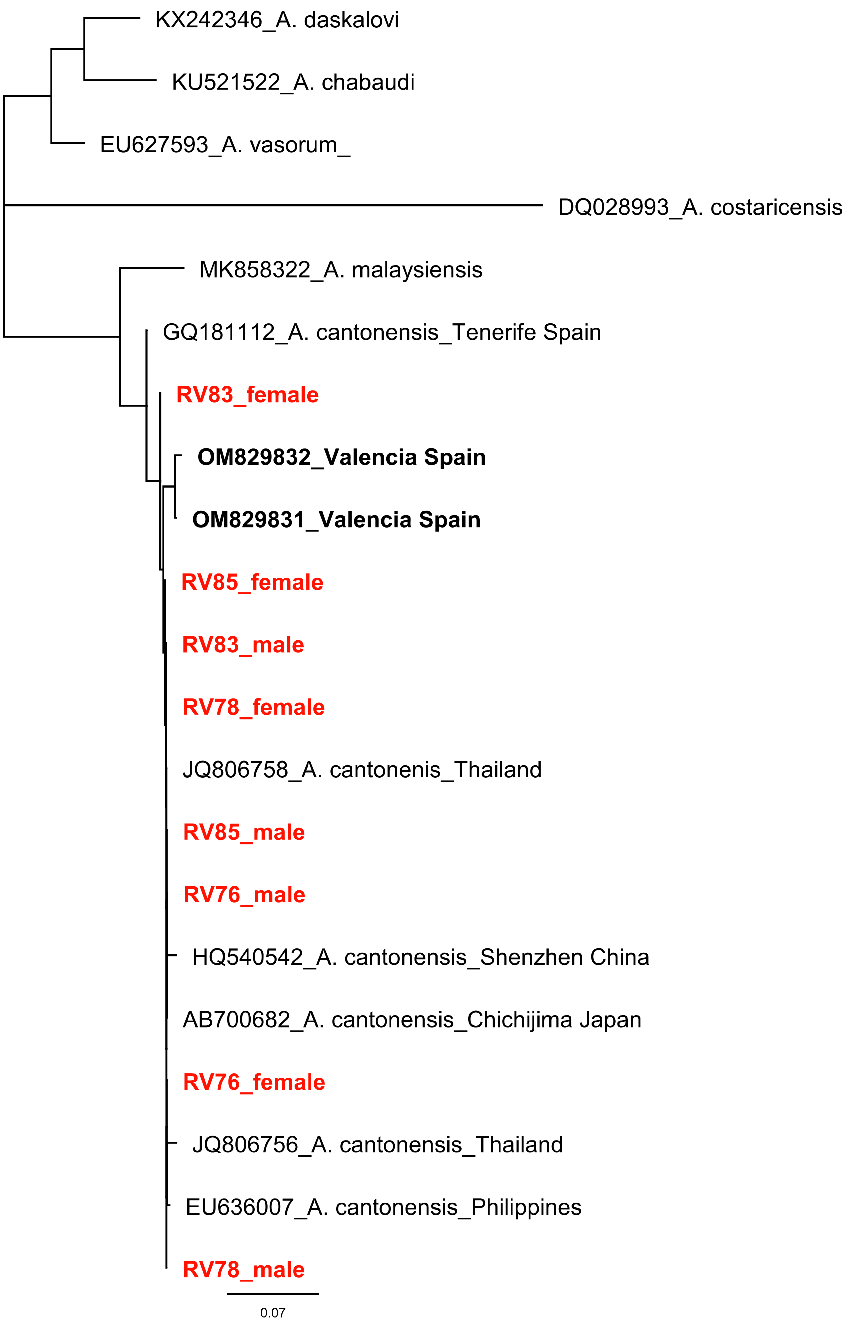

Figure S2. Neighbor-Joining phylogenetic tree of *Angiostrongylus cantonensis*, *A. malaysiensis*, *A. costaricensis*, *A. vasorum*, *A. chabaudi*, and *A. daskalovi* based on the partial ITS2 sequences. Bold letters indicate sequences obtained in our previous study {15] and red letters are sequences obtained in our present study.

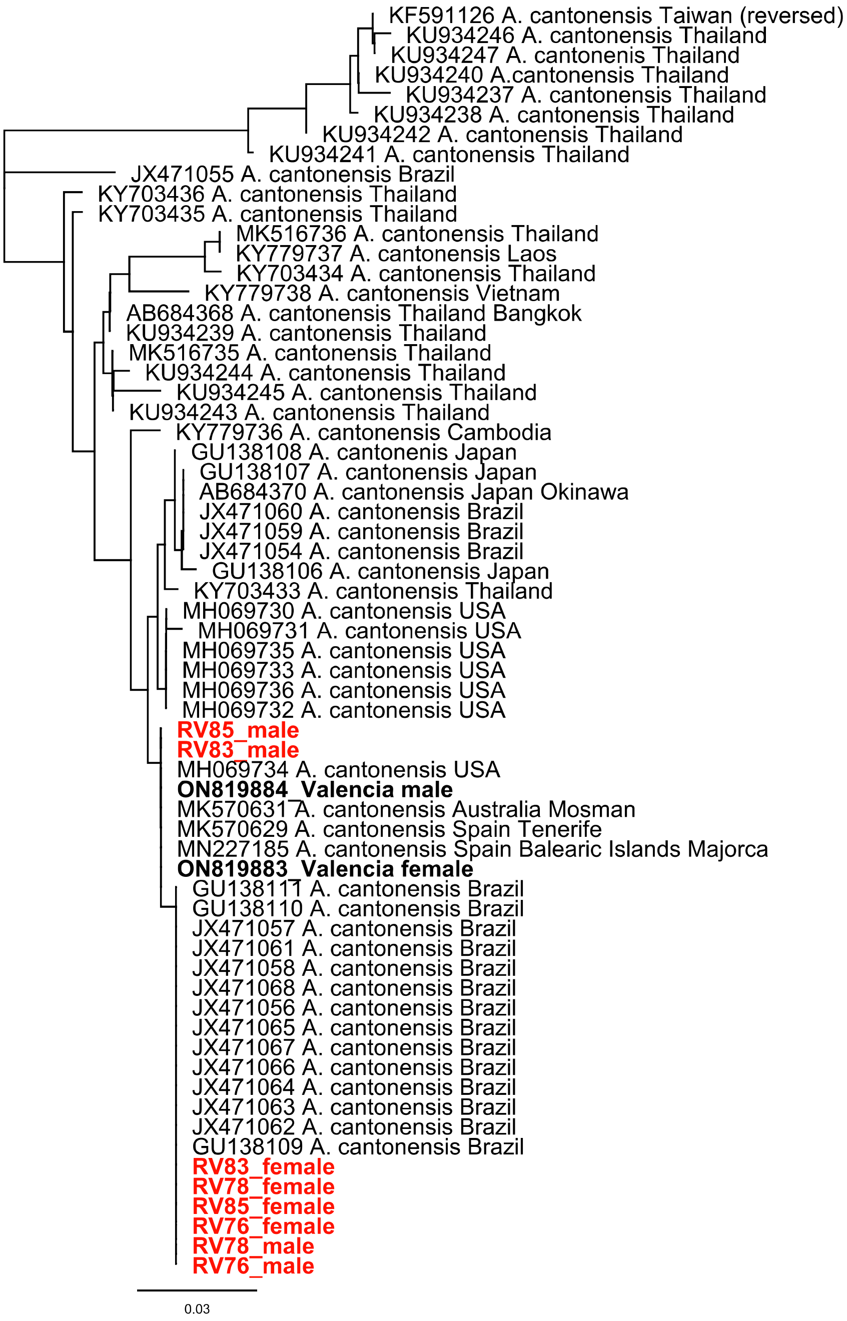

Figure S3. Neighbor-Joining phylogenetic tree of *Angiostrongylus cantonensis* geographical isolated individuals based on the partial COI sequences. Bold letters indicate sequences obtained in our previous study [15] and red letters are sequences obtained in our present study.

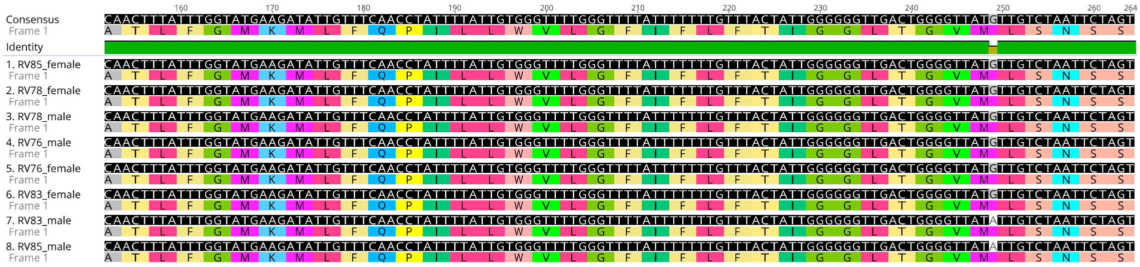

Figure S4. ClustalW alignment performed with partial COI sequences from RV76 female, RV76 male, RV83 female, RV78 female, RV78 male, RV85 male, RV83 male, and RV85 female samples.
